# Targeting SHP‐1‐Mediated Inhibition of STAT3 and ERK Signalling Pathways Rescues the Hyporesponsiveness of MHC‐I‐Deficient NK‐92MI

**DOI:** 10.1111/cpr.70035

**Published:** 2025-04-01

**Authors:** Kuo Yu, Xiaolong Liu, Guangyuan Wu, Zhongyao An, Xin Wang, Yang Liu, Hailong Wang, Mingli Huang, Linlin Zhao, Ce Shi, Xin Sun, Lu Xu, Sen Qi, Xin Zhang, Yueqiu Teng, Song Guo Zheng, Zhiren Zhang, Zhenkun Wang

**Affiliations:** ^1^ NHC Key Laboratory of Cell Transplantation Harbin Medical University Harbin China; ^2^ Central Laboratory First Affiliated Hospital, Harbin Medical University Harbin China; ^3^ ZKcell Biotechnology (Heilongjiang) Co., Ltd Harbin China; ^4^ Obstetrical Department First Affiliated Hospital, Harbin Medical University Harbin China; ^5^ Department of Blood Transfusion First Affiliated Hospital, Harbin Medical University Harbin China; ^6^ Department of Pathology First Affiliated Hospital, Harbin Medical University Harbin China; ^7^ Department of Immunology School of Cell and Gene Therapy, Songjiang Research Institute, Shanghai Songjiang District Central Hospital, Shanghai Jiaotong University School of Medicine Shanghai China; ^8^ Department of Cardiology and Pharmacy and Breast Cancer Surgery Harbin Medical University Cancer Hospital, Institute of Metabolic Disease, Heilongjiang Academy of Medical Science, Heilongjiang Key Laboratory for Metabolic Disorder and Cancer Related Cardiovascular Diseases Harbin China; ^9^ Department of Hematology First Affiliated Hospital of Harbin Medical University, the Institute of the Hematology and Oncology of Heilongjiang Province Harbin China; ^10^ The Somatic Cells Bioengineering Technology Research Center of Qinhuangdao Qinhuangdao China

**Keywords:** ERK1/2, MHC‐I, NK cells, SHP‐1, STAT3

## Abstract

Natural Killer (NK) cells have shown promising prospects in ‘off‐the‐shelf’ cell therapy, particularly the NK‐92 cell line, which can serve as a foundation for the next generation of universal chimeric antigen receptor (CAR)‐engineered NK products. A key strategy for generating universal cellular products is the elimination of the beta‐2‐microglobulin (B2M) gene, which encodes a component of MHC class I molecules (MHC‐I) that plays a role in the presentation of foreign antigens and in the ‘licensing’ or ‘education’ of NK cells. To functionally study the impacts of MHC‐I deficiency on NK‐92, we generated a B2M knockout (KO) NK‐92MI (B‐92) cell line and compared the multidimensional properties of B2M KO and wild‐type NK‐92MI cells in terms of biological phenotypes, effector functions, and transcriptomic signatures. We observed a decrease in activating receptors, cytokine production, and cytotoxicity in B‐92 cells. Further analysis of signalling events revealed that the upregulated expression and phosphorylation of SHP‐1 in B‐92 cells inhibited the phosphorylation levels of STAT3 and ERK, thereby affecting their killing function. By knocking out SHP‐1 (PTPN6), we partially restored the cytotoxic function of B‐92 cells. Notably, we also found that CAR modification can overcome the hyporesponsiveness of B‐92 cells. These findings will facilitate further exploration in the development of NK cell‐based products.

## Graphical abstract

B2M knockout (KO) represents a crucial strategy for generating universal NK‐92MI cell products. This approach results in increased phosphorylation of SHP‐1 and inhibited phosphorylation of STAT3 and ERK, which ultimately affect their cytotoxic capabilities. SHP‐1 KO facilitates the restoration of cytotoxic function in B2M KO NK‐92MI cells.

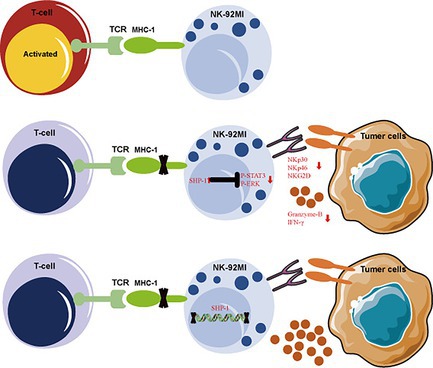

## 1 Introduction

Immune cell therapies have gained considerable momentum in the past few years, and natural killer (NK) cell‐based immunotherapies are attracting increasing interest as a promising platform for developing off‐the‐shelf products [1, 2]. However, NK cells derived from blood face several challenges, including limited expansion capacity, difficulties in genetic modification, and high costs. The NK‐92MI cell line represents an immortalised cell line that displays standard NK cell phenotypes and characteristics, possessing the ability to identify tumour cells and virus‐infected cells. In addition to releasing perforin and granzyme to eliminate target cells, NK‐92MI cells enhance the activation of other immune cells by synthesising cytokines [3, 4]. Trials have shown that the utilisation of low‐dose irradiated NK‐92MI cells in adoptive cell transfer (ACT) therapy is safe and exhibits encouraging potential for treating specific types of tumours [5, 6].

With the advancement of gene‐edited immune cell therapy technology, methods such as chimeric antigen receptor (CAR) modification have led to groundbreaking improvements in the treatment of blood cancers [7]. Compared to personalised CAR‐T therapy, CAR‐modified NK‐92MI cell therapy (CAR‐NK‐92MI) offers advantages such as straightforward gene editing, consistent gene modifications, high‐volume production capabilities, and a reduced risk of cytokine release syndrome (CRS) [8, 9]. Through mild radiation treatment (approximately 10 Gy), CAR‐NK‐92MI cells lose their expansion capacities while maintaining their short‐term destructive capabilities against tumour cells [10, 11]. In animal experiments, CAR‐NK‐92MI effectively suppressed tumour growth and enhanced survival rates across various tumour models [12, 13]. These findings indicate that CAR‐NK‐92MI cells could serve as a versatile and more efficient cellular product, showing potential for innovative advancements in cancer immunotherapy for malignancies.

In adoptive immunotherapy, the use of allogeneic cells that express antigenic properties raises concerns about immune rejection [14, 15]. Consequently, the development of universal cell products must prioritise addressing the issue of immune rejection. Given that B2M is a crucial subunit of MHC‐I, B2M knockout (KO) presents a convenient and effective strategy for generating universal cell products [16, 17, 18]. This approach results in the absence of all MHC‐I molecules on the cell surface, thereby preventing immune recognition by cytotoxic CD8^+^ T cells and effectively mitigating the challenge of immune rejection in allogeneic cell transplants [19, 20, 21, 22].

In this study, we generated a B2M KO NK‐92MI (B‐92) cell line. However, we observed that B2M KO led to a significant decrease in the killing efficiency of NK‐92MI cells against K562 and NALM6 cells. To investigate the underlying mechanisms, we employed RNA sequencing to identify key signalling pathways that influence NK‐92MI function. Our findings demonstrate that the knockout of B2M results in an upregulation of SHP‐1 and phosphorylated SHP‐1 expression levels in NK‐92MI cells, which subsequently inhibits the phosphorylation of STAT3 and ERK1/2. This signalling cascade leads to the downregulation of activating receptors on the cell surface, significantly impairing the cytotoxic capabilities of NK‐92MI cells. Furthermore, by knocking out PTPN6, we have partially restored their cytotoxic function, and its hyporesponsiveness can also be addressed by CAR modification, allowing NK‐92MI cells to maintain low immunogenicity while preserving their inherent cytotoxicity.

## 2 Materials and Methods

### 2.1 Cell Lines

The NK‐92MI, K562, NALM6, and 293 T cell lines used in this study are maintained at the Laboratory of Haematology‐Oncology Centre of the First Affiliated Hospital of Harbin Medical University. NK‐92MI cells were incubated in Lymphocyte Serum‐free Medium, KBM 581 (88‐581‐CM; Corning). K562 and NALM6 cells were cultured in RPMI‐1640 medium (11,875,093; Gibco) supplemented with 10% fetal bovine serum (FBS; F0193; Sigma). 293 T cells were cultured in Dulbecco's modified Eagle medium (DMEM; 11,965,118; Gibco) supplemented with 10% FBS (Gibco).

### 2.2 Plasmid Construction

Exon sequences of the human B2M gene and human PTPN6 gene were retrieved from the Ensembl database (http://useast.ensembl.org/index.html) and sgRNA primers targeting the first exon were designed using the CRISPOR website (http://crispor.tefor.net/crispor). The lentiviral vector employed in this study was lentiCRISPR v2 (Addgene, #52961). The vectors were constructed using traditional molecular cloning methods, including PCR, oligo annealing, restriction enzyme cleavage, ligation. Custom oligonucleotides were obtained from Genewiz (Suzhou, China). These vectors were then transfected into *E. coli* strain Stabl3 competent cells and screened using ampicillin. The fidelity of the vector sequences was validated by Sanger sequencing. In the rescue experiment following B2M KO, a silent mutation that does not change the amino acid sequence was introduced in the sgRNA target‐adjacent PAM sequence. The B2M overexpression sequence was synthesized and then cloned into the PLVX lentiviral vector, with the EGFRt reporter tag linked via an IRES for coordinated co‐expression. Transduced cells were then enriched through FACS using anti‐EGFR monoclonal antibodies.

All primer sequences:

**Table:** 

*PTPN6*‐sgRNA‐F	CACCGTCACGCACAAGAAACGTCCA
*PTPN6‐*sgRNA‐R	AAACTGGACGTTTCTTGTGCGTGAC
*B2M*‐sgRNA‐F	CACCGGCCGAGATGTCTCGCTCCG
*B2M*‐sgRNA‐R	AAACCGGAGCGAGACATCTCGGCC

### 2.3 Construction of CD19‐CAR NK‐92MI Cells

The CD19‐CAR (FMC63 scFv, 4‐1BB and CD3ζ intracellular domain) lentivirus solution was purchased from GENECHEM (Shanghai, China). Viral infection was conducted in 96‐well plates with 5 × 10^4^ NK‐92MI cells in 100 μL of lentivirus solution (MOI: 10). Cells were centrifuged at 1000 × relative centrifugal field (RCF) at 32°C for 60 min and subsequently incubated at 37°C for 12 h. Approximately 96 h after transfection, CD19‐CAR positive cells were sorted as CD19‐CAR‐NK‐92 cells by BD FACS Aria II.

### 2.4 Flow Cytometry

For membrane staining, 2 × 10^5^ cells were collected and washed with DPBS. Subsequently, the corresponding antibodies were added to the cells, followed by incubation at 4°C for 30 min. After washing, the cells were then resuspended in 200 μL of DPBS. Flow cytometry (Accuri C6; BD) was utilised to analyse the samples.

For intracellular cytokine staining, 5 × 10^5^ NK‐92MI cells were plated and treated with the intracellular protein transport inhibitor BFA (347,688; BD). K562 cells and NK‐92MI cells were co‐cultured for 5 h to activate NK‐92MI. Following the addition of CD56 antibody, the samples underwent a 30‐min incubation. After centrifugation, the supernatant was discarded, and the cells were treated with a fixation solution (554,722; BD) and incubated for an additional 40 min. Post‐centrifugation, 200 μL of permeabilization buffer (554,722; BD) was added. Subsequently, the cells were incubated in the dark with IFN‐γ and Granzyme‐B antibodies for 30 min. After two washes with permeabilization buffer (554,722; BD), the cells were resuspended in 200 μL of DPBS. Flow cytometry was then employed to analyse the samples.

### 2.5 Western Blotting

For Western blot analysis, wild‐type and gene‐edited NK‐92MI cells were lysed in RIPA lysis buffer (P0013B, Beyotime) containing protease inhibitors on ice for 30 min. The lysates were then centrifuged at 12,000 × RCF for 25 min, and the resulting supernatants were collected. Protein concentrations were quantified using the BCA protein assay kit (P0009B, Beyotime). Equal quantities of protein were loaded, separated on a sodium dodecyl sulfate‐polyacrylamide gel electrophoresis system, and subsequently transferred to a PVDF membrane (IPVH00010; Millipore). Following a 2‐h blocking step with 5% skim milk at room temperature, the membrane was incubated with the primary antibody overnight at 4°C. The immunoblots were visualised using HRP‐conjugated secondary antibodies. The primary antibodies used included: SHP‐1 (P81718, Immunoway), Phospho‐SHP‐1 (Tyr564) (YP0145, Immunoway), STAT3 (#4904, Cell Signalling), Phospho‐STAT3 (Tyr705) (#9145, Cell Signalling), ERK1/2 (YT1625, Immunoway), Phospho‐ERK1/2 (Thr202/Y204) (YP1197, Immunoway), GAPDH (P04406, Abways).

### 2.6 Cytotoxicity Assay

NK‐92MI and target cells were co‐cultured under comparable conditions at varying effector‐to‐target (*E:T*) ratios in a 96‐well plate for 20 h. The samples were analysed using the Cellcyte X microscope (Cytena, Germany) along with the associated Cellcyte software.

### 2.7 RNA Sequencing (RNA‐Seq) and Data Analysis

High‐throughput sequencing was performed by Genewiz Biotechnology LLC (Suzhou, China). Total RNA was employed as the input material for RNA sample preparation. Briefly, poly(T) oligonucleotides were attached to magnetic beads to separate mRNA and total RNA. After the purification of PCR products with g Oligo(dT) beads, the quality of the library was evaluated using the Cutadapt (v1.9.1) system. Subsequently, differential expression analysis between the two groups was carried out using the DESeq2 Bioconductor package (v1.20.0). Genes showing significant differential expression (DEGs) were defined by a Fold Change > 1.0 and an adjusted *p* value < 0.05. KEGG and Gene Ontology (GO) enrichment analyses were conducted using the GOSeq (v1.34.1) package. Additionally, Gene Set Enrichment Analysis (GSEA, v4.3.2) was utilised to explore the functional enrichment of genes and pathways.

### 2.8 In Vivo Animal Models and Treatments

NCG mice were purchased from GemPharmatech (Nanjing, China) and maintained in a sterile environment for 1 week to allow for acclimatisation. Twenty‐four hours after administering Busulfan at a dosage of 10 mg/kg, transplant 1 × 10^5^ NALM6 cells into the NCG mice (6‐8 weeks old, female) via tail vein injection. Two days later, 6 groups of 5 Gy irradiated effector cells were injected respectively, and 1 × 10^7^ effector cells were injected into the tail vein of each mouse three times a week for a total of 3 weeks. Mice were injected with D‐luciferin (Gold Biotechnology, USA) at a dose of 150 mg/kg on day 14, day 21, and day 28, and imaged using IndiGo (BERTHOLD, Germany). Protocols were approved by the Harbin Medical University Animal Care and Use Committee guideline.

### 2.9 Statistical Analysis

Statistical analyses were conducted using GraphPad Prism version 9.5.1. The significance of differences between two groups was assessed with an unpaired Student's *t*‐test, while comparisons involving more than two groups were performed using One‐way ANOVA. Two‐way ANOVA analysis was employed for the statistical analysis of multifactor variance. Significance levels are indicated as follows: *p < 0.05, **p < 0.01, ***p < 0.001, ****p < 0.0001 and ns for *p* > 0.05.

## 3 Results

### 3.1 Generation of B2M‐KO‐NK‐92MI Cells

**FIGURE 1:**
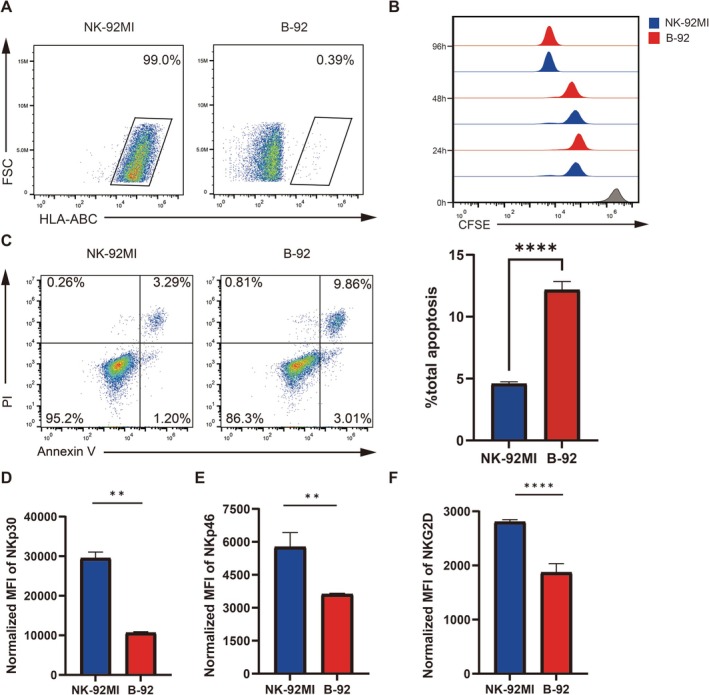
Construction of B2M KO NK‐92MI. (A) Flow cytometric analysis was performed to evaluate the expression of HLA‐ABC on the surface of B‐92 cells. (B) The proliferation capabilities of NK‐92MI and B‐92 cells were assessed using flow cytometry over a 96‐h period. (C) Apoptosis levels in both NK‐92MI and B‐92 cells were also measured through flow cytometric detection. The analysis included the detection of activating receptors, specifically (D) NKp30, (E) NKp46, and (F) NKG2D, with *n* = 3 for each measurement. Statistical analysis was conducted using the *t*‐test, and the data are presented as mean ± standard deviation (SD). Significance levels are indicated as follows: *p < 0.05, **p < 0.01, ***p < 0.001, ****p < 0.0001 and ns for *p* > 0.05.

We successfully generated and separated the B‐92 cells (Figure 1A), stained NK‐92MI and B‐92 cells with CFSE dye, and cultured them under standard conditions for 96 h. CFSE dilution was assessed by flow cytometry at 24‐h intervals. The results indicated that B2M KO did not affect the proliferation of NK‐92MI cells (Figure 1B). Notably, B‐92 cells displayed a higher level of spontaneous apoptosis compared to NK‐92MI cells (Figure 1C). To evaluate the influence of B2M knockout on the phenotype of NK‐92MI cells, we analysed the expression of activating receptors. Flow cytometry analysis revealed that the expressions of NKp30, NKp46, and NKG2D were significantly downregulated in B‐92 cells compared to wild‐type NK‐92MI cells (Figure 1D–F).

### 3.2 B2M Knockout Decreases the Cytotoxicity of NK‐92MI Cells Against Cancer Cells

**FIGURE 2:**
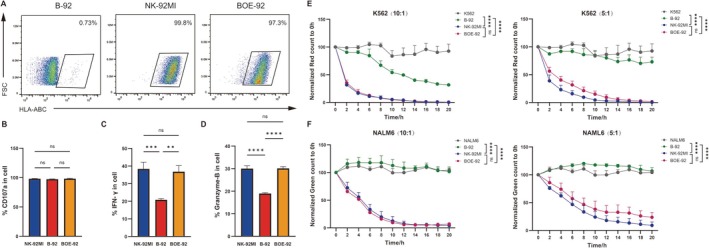
The degranulation capacity, cytokine synthesis levels, and cytotoxicity of B‐92 cells. (A) Flow cytometric detection of the expression levels of HLA‐ABC on the surface of B‐92, NK‐92MI, BOE‐92 cells. (B) Flow cytometric analysis of degranulation marker CD107a in B‐92, NK‐92MI, and BOE‐92 cells. (C) Flow cytometric analysis of intracellular IFN‐γ level in B‐92, NK‐92MI, and BOE‐92 cells. (D) Flow cytometric analysis of granzyme B expression in B‐92, NK‐92MI, and BOE‐92 cells, with *n* = 3 for each measurement. Statistical analysis was performed using the t‐test, and the data are presented as mean ± standard deviation (SD). Using the Cellcyte X, we continuously monitored the cytotoxic effects of B‐92, NK‐92MI, and BOE‐92 on (E) K562 and (F) NALM6 for 20 h under varying *E:T* ratios, with *n* = 3 for each measurement. Statistical analysis was performed using Two‐way ANOVA. Data statistics are presented as mean ± standard deviation (SD). Significance levels are indicated as follows: *p < 0.05, **p < 0.01, ***p < 0.001, ****p < 0.0001, and ns for *p* > 0.05.

To further explore the effect of B2M gene knockout on the function of NK‐92MI, we assessed the degranulation capacity, cytokine synthesis levels, and cytotoxicity of B‐92 cells. Notably, in contrast to NK‐92MI cells, B‐92 cells exhibited significantly lower levels of IFN‐γ and Granzyme B production, while CD107a expression levels showed no significant difference. However, IFN‐γ and Granzyme B levels were restored when we overexpressed the B2M gene in B‐92 cells (BOE‐92) (Figure 2A–D). To further evaluate the cytotoxicity of B‐92, we selected two distinct haematological cancer cell lines as target cells: the myelogenous leukaemia cell line K562 and the human B lymphoblastic leukaemia cell line NALM6. NK‐92MI or B‐92 cells were co‐cultured with each target cell type under identical conditions at varying *E:T* ratios (10:1, 5:1) in a 96‐well plate for 20 h. The results indicate that following the knockout of the B2M gene, the cytotoxic efficiency of NK‐92MI cells significantly declined. As anticipated, at both the 10:1 and 5:1 *E:T* ratios, no significant difference was observed between BOE‐92 and NK‐92MI (Figure 2E,F). Hence, it can be affirmed that the knockout of B2M decreases the cytotoxic function of NK‐92MI cell lines.

### 3.3 B2M Knockout Reduced the Phosphorylation of STAT3 and ERK Mediated by SHP‐1

**FIGURE 3:**
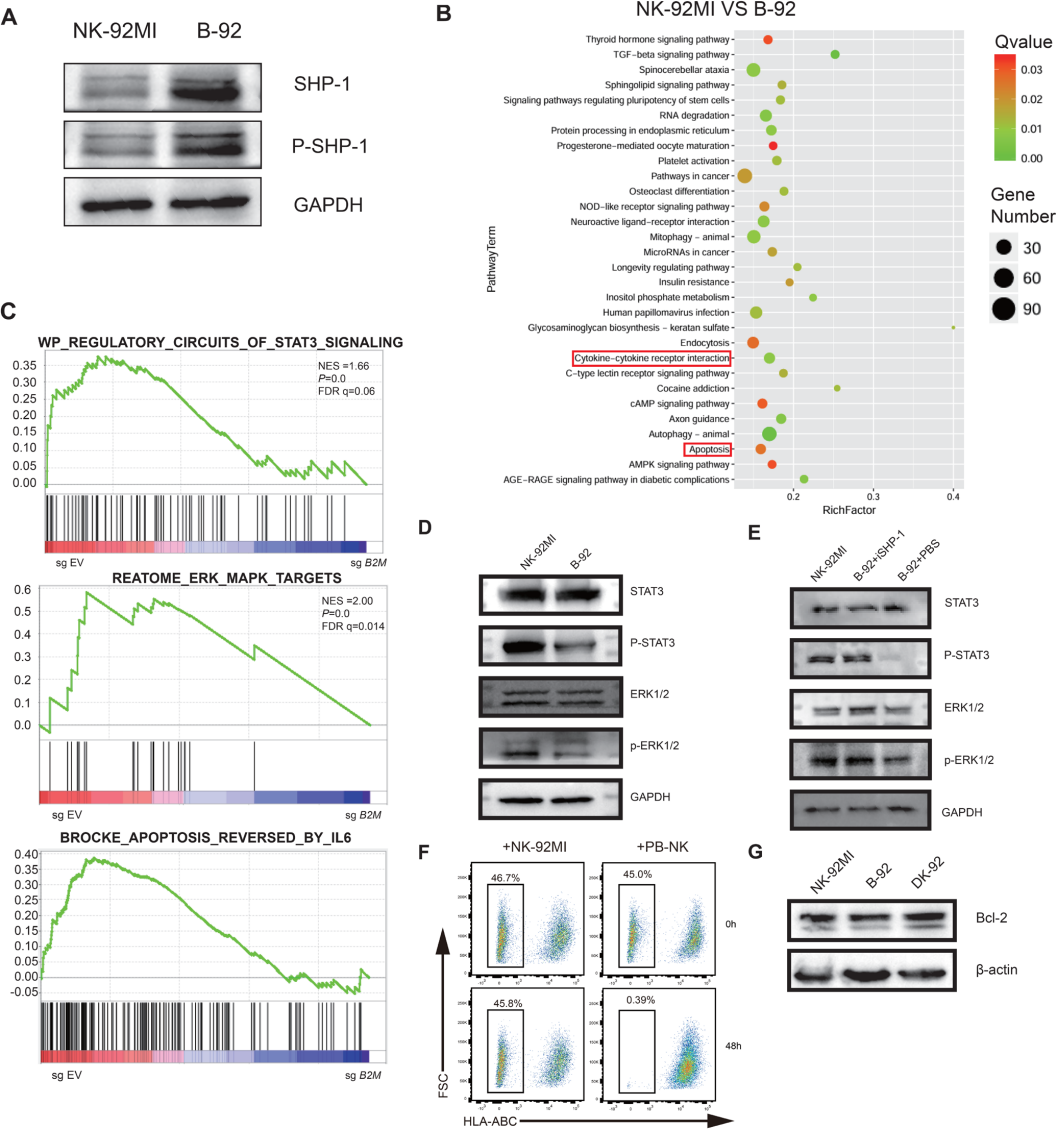
RNA‐seq analysis of NK‐92MI cells post‐KO of B2M and Western blot validation. (A) Western blot analysis shows the expression levels of SHP‐1 and phosphorylated SHP‐1 in both NK‐92MI and B‐92 cells. (B) The scatter plot illustrating differential gene KEGG enrichment displays pathway names on the vertical axis, while the horizontal axis represents the Rich factor. (C) GSEA signature enrichment plots compare SgEmpty Vector (SgEV) and SgB2M, with the normalised enrichment score (NES) indicated. (D) Western blot analysis shows the expression levels of STAT3, phosphorylated STAT3, ERK, and phosphorylated ERK in NK‐92MI and B‐92 cells. (E) Western blot analysis demonstrated that after pretreatment with the SHP‐1 inhibitor, the expression levels of STAT3, phosphorylated STAT3, ERK, and phosphorylated ERK were evaluated in the NK‐92MI and B‐92 cell lines. (F) Flow cytometric detection of the expression levels of HLA‐ABC in the group of NK‐92MI and the group of PB‐NK. (G) Western blot analysis shows the expression levels of Bcl‐2 in NK‐92MI, B‐92, and DK‐92 cells.

Recent studies have demonstrated that in mouse models lacking MHC‐I, the inhibitory receptors of ‘educated’ NK cells sequester SHP‐1 from the immunological synapse, resulting in increased levels of free SHP‐1 [23]. We hypothesize that the excessive activation of SHP‐1 contributes to the suppression of activating receptors in B‐92 cells. Consequently, we measured the protein expression levels of SHP‐1 and phosphorylated SHP‐1 (Tyr564) in the NK‐92MI and B‐92 cells. In comparison with NK‐92MI, the expression levels of SHP‐1 and phosphorylated SHP‐1 in B‐92 cells were found to be elevated (Figure 3A). To confirm whether the upregulation of SHP‐1/p‐SHP‐1 resulted in a decrease in NK‐92MI cell cytotoxicity, we conducted RNA‐seq analysis of B‐92 cells. The enrichment analysis of the KEGG database revealed that the knockout of B2M impacts biological pathways related to cell metabolism, immune response, and autophagy, notably including the cytokine‐cytokine receptor interaction pathway and the apoptosis pathway (Figure 3B). Through gene set enrichment analysis (GSEA), we identified significant enrichment in the STAT3/ERK pathways and STAT3‐related pro‐apoptotic pathways following B2M KO (Figure 3C). In the cytotoxic signalling pathway of NK cells, ERK and STAT3 are positioned downstream of SHP‐1, with their phosphorylation levels being crucial for pathway activation [24, 25]. We assessed the expression levels of ERK1/2, STAT3, phosphorylated ERK1/2 (Thr202/Tyr204), and phosphorylated STAT3 (Tyr705) in NK‐92MI and B‐92 cells. The results revealed a significant decrease in the tyrosine phosphorylation levels of ERK1/2 and STAT3 in B‐92 cells (Figure 3D). Subsequently, we utilised the SHP‐1 specific inhibitor (NSC‐87877; Selleck) to pretreat B‐92 cells, thereby validating the regulatory effect of SHP‐1 on STAT3 and ERK (Figure 3E).

To further confirm that B‐92 apoptosis is induced by the STAT3 inhibition rather than fratricide, we co‐cultured B‐92 with NK‐92MI or peripheral blood NK cells (PB‐NK). Surprisingly, PB‐NK almost completely eliminated B‐92 cells within 48 h, while NK‐92MI did not kill B‐92 cells (Figure 3F). This finding suggests a difference in recognition patterns between NK‐92MI and peripheral blood NK cells. Since NK‐92 cells lack most of the receptors in the KIR family, the ‘missing‐self’ theory may not apply to NK‐92 cells. This finding is consistent with earlier studies indicating that NK‐92‐mediated cytolysis differs partially from that of PB‐NK. Specifically, NK‐92 can kill most MHC‐I‐positive tumour cells but exhibits poor killing of MHC‐I‐deficient K562 cells [26]. Subsequently, we assessed the expression of the anti‐apoptotic protein Bcl‐2, which is mainly regulated by the STAT3 pathway. Bcl‐2 expression decreased in B‐92 cells (Figure 3G).

These findings indicated that the knockout of the B2M gene in NK‐92MI cells led to an upregulation of SHP‐1 expression and phosphorylation, resulting in the inhibition of ERK1/2 and STAT3 phosphorylation. Consequently, this downregulated the synthesis of IFN‐γ and the release of granzyme B in NK‐92MI cells, ultimately affecting their cytotoxic function. Notably, STAT3 serves as a crucial regulator for activation by NKG2D, NKp30, and NKp46 [27, 28]; the inhibition of these activating receptors might be attributed to the suppression of STAT3 phosphorylation. Furthermore, the inhibition of STAT3 signalling induces apoptosis and decreases anti‐apoptotic protein Bcl‐2.

### 3.4 Knockout of the PTPN6 Gene Rescues the Cytotoxic Function of B‐92

**FIGURE 4:**
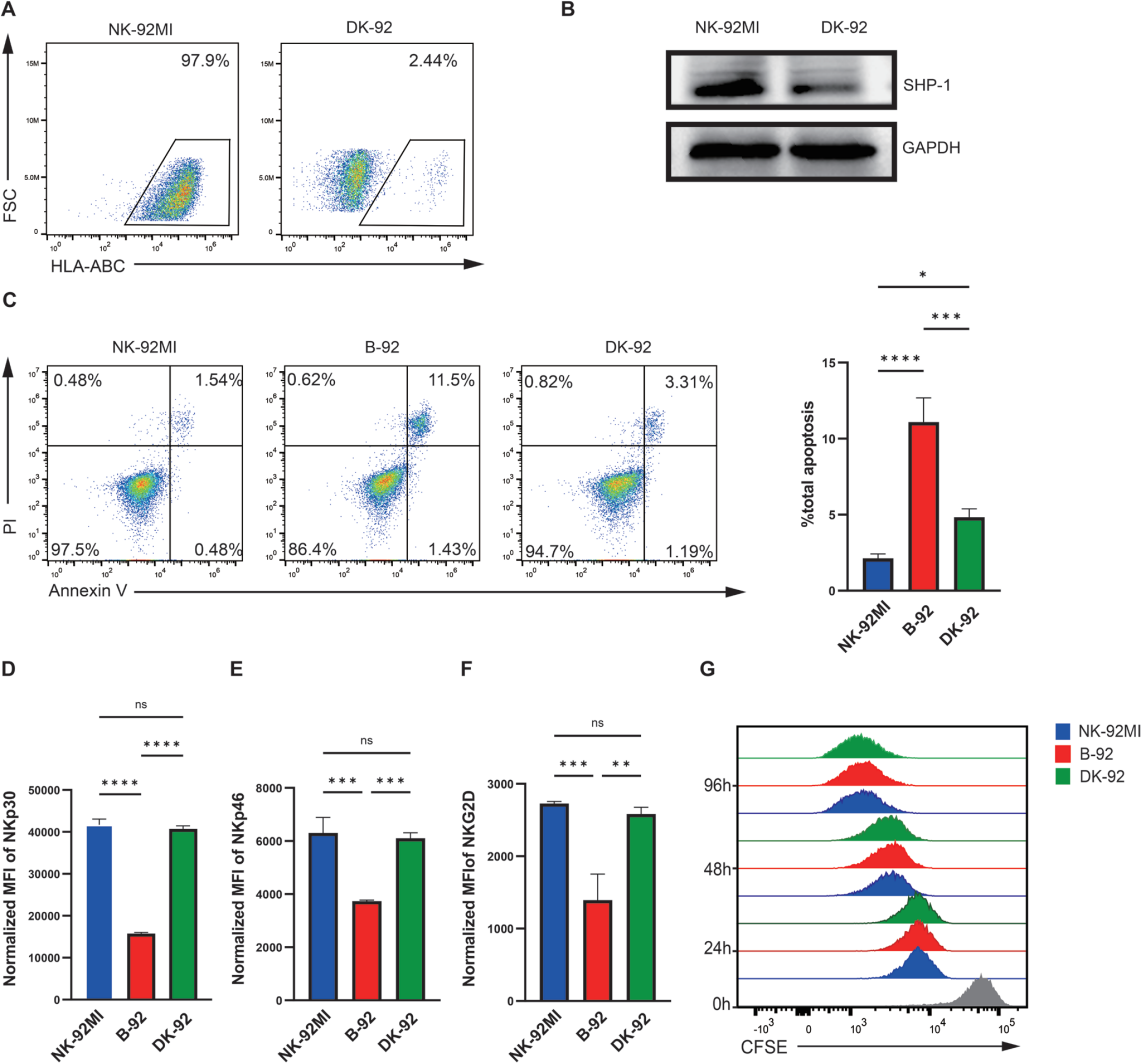
Construction of DK‐92 and assessment of their biological functions. (A) Flow cytometric analysis of HLA‐ABC expression on the surface of DK‐92 cells. (B) Western blot analysis showed the knockout efficiency of SHP‐1. (C) Flow cytometric detection of apoptosis levels in NK‐92MI, B‐92, and DK‐92 cells. Flow cytometric analysis of activating receptors (D) NKp30, (E) NKp46, and (F) NKG2D, with *n* = 3 for each measurement. (G) The proliferation capabilities of NK‐92MI, B‐92, and DK‐92 cells were assessed using flow cytometry over a 96‐h period. The statistical method used was one‐way analysis of variance, with data presented as ± standard deviation (SD). Significance levels are indicated as follows: *p < 0.05, **p < 0.01, ***p < 0.001, ****p < 0.0001, and ns for *p* > 0.05.

To further evaluate whether SHP‐1 is a viable target for rescuing the phenotype and cytotoxic function of B‐92, we generated a PTPN6 and B2M double knockout NK‐92MI cell line (Figure 4A,B). We first assessed the level of apoptosis in PTPN6/B2M‐KO‐NK‐92MI cells (DK‐92). The results indicated that the level of apoptosis in DK‐92 cells was significantly decreased compared to B‐92 cells (Figure 4C), and Bcl‐2 expression in DK‐92 cells also recovered (Figure 3G). Additionally, the expression levels of NKp30, NKp46, and NKG2D were significantly increased in DK‐92 compared to B‐92 cells, with no significant difference observed when compared to NK‐92MI cells (Figure 4D–F). Besides that, there was no difference in the proliferation of B‐92, NK‐92, and DK‐92 cells (Figure 4G).

**FIGURE 5:**
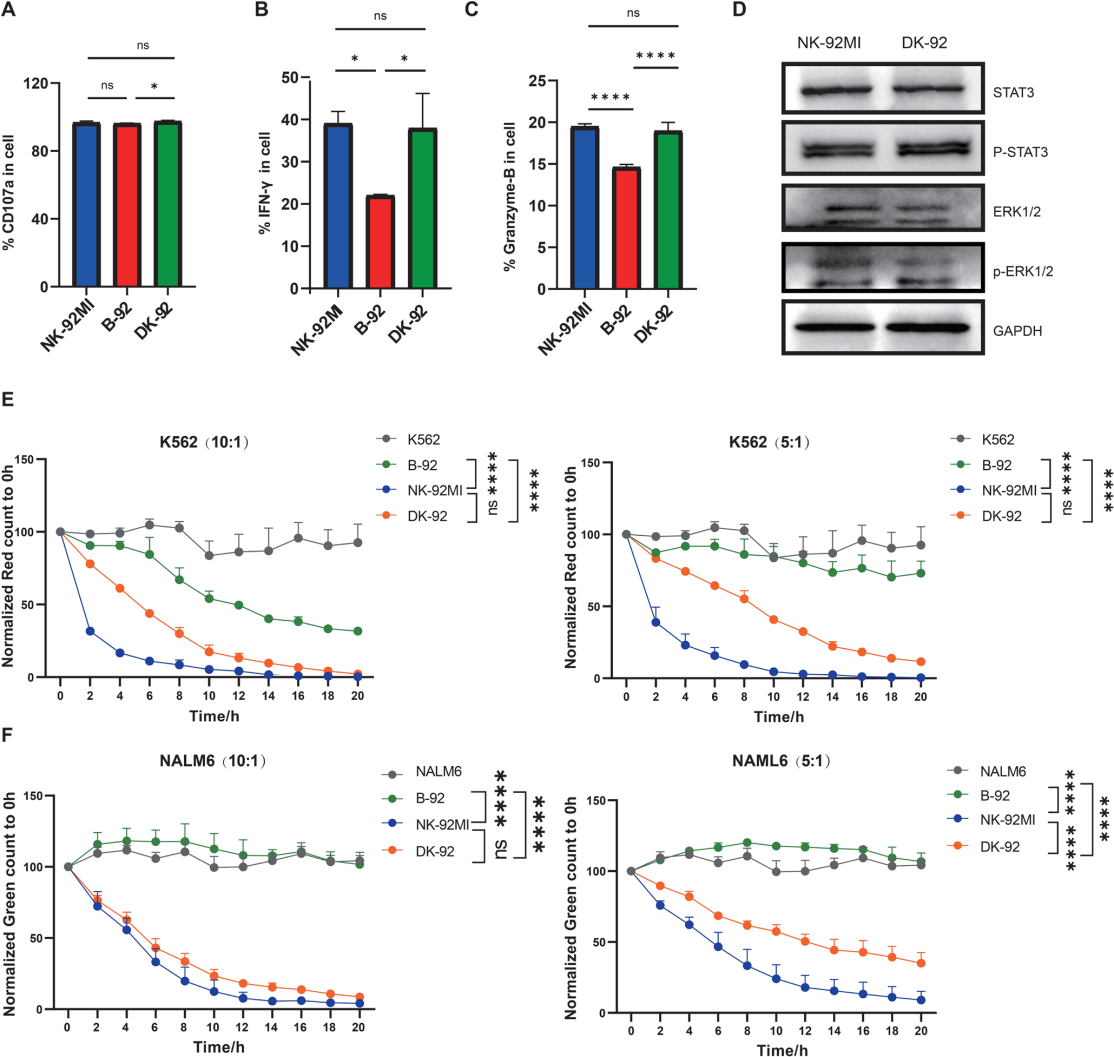
Detection results of the cytotoxic function in DK‐92. (A) Flow cytometric analysis of the degranulation marker CD107a in NK‐92MI and DK‐92 cells. (B) Flow cytometric analysis of the intracellular IFN‐γ levels in NK‐92MI and DK‐92 cells. (C) Flow cytometric analysis of granzyme B expression in NK‐92MI and DK‐92 cells. (D) Western blot analysis showed the expression levels of STAT3, phosphorylated STAT3, ERK, and phosphorylated ERK in NK‐92MI and DK‐92 cells. Utilising the Cellcyte X, we continuously monitored the cytotoxic effects of NK‐92MI and DK‐92 cells on (E) K562 and (F) NALM6 cells for 20 h under different *E:T* ratios, with *n* = 3 for each measurement. Statistical analysis was performed using the Two‐way ANOVA. Data statistics are presented as mean ± standard deviation (SD). Significance levels are indicated as follows: *p < 0.05, **p < 0.01, ***p < 0.001, ****p < 0.0001, and ns for *p* > 0.05.

Subsequently, NK‐92MI, B‐92, and DK‐92 cells were co‐cultured with K562 and NALM6 cells under identical conditions at varying *E:T* ratios (10:1, 5:1) for 20 h. Notably, DK‐92 demonstrated a significant restoration of cytotoxic functionality compared to B‐92 (Figure 5A–C). We also measured the phosphorylation levels of ERK1/2 and STAT3 in DK‐92 cells and observed no significant differences compared to those in NK‐92MI cells (Figure 5D). Furthermore, our findings confirmed that SHP‐1 knockout could partially restore the cytotoxicity of B‐92 cells (Figure 5E,F).

### 3.5 The CAR‐Modified B‐92 Cells Can Overcome the Issue of Reduced Cytotoxicity

**FIGURE 6:**
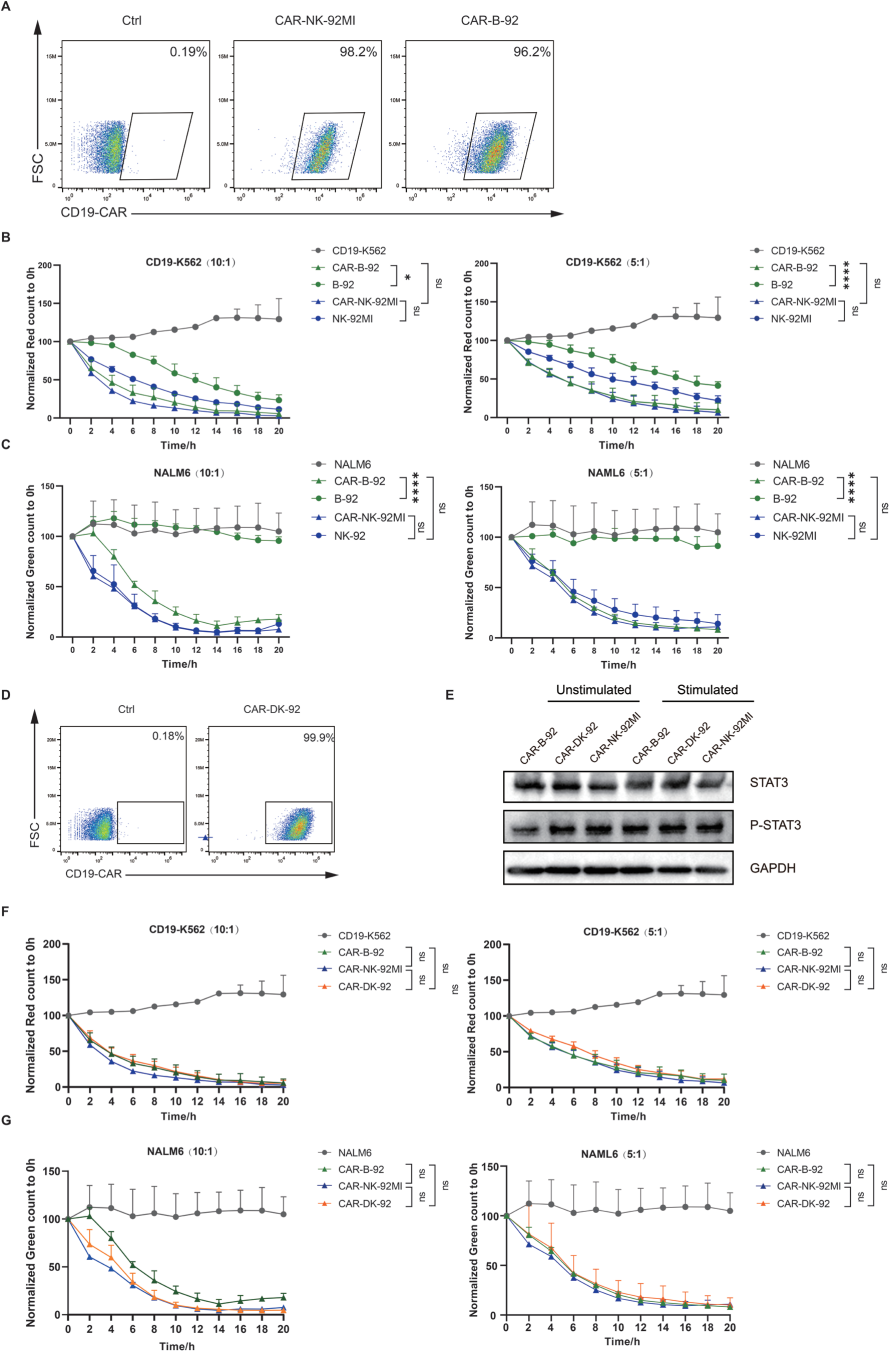
The cytotoxic efficiency of NK‐92MI, B‐92, and DK‐92 against K562 and NALM6 cells following CD19‐CAR modification. (A) Flow cytometric detection of the expression levels of CD19‐CAR on the surface of NK‐92MI and B‐92 cells. Using the Cellcyte X, we continuously monitored the cytotoxic effects of CAR‐NK‐92MI and CAR‐B‐92 on (B) CD19‐K562 and (C) NALM6 cells for 20 h under different *E:T* ratios. *n* = 3. (D) Flow cytometric detection of the expression levels of CD19‐CAR on the surface of CAR‐DK‐92 cells. (E) Western blot analysis demonstrated that after stimulation with the CD19‐antigen, the expression levels of STAT3 and phosphorylated STAT3 were evaluated in the CAR‐NK‐92MI, CAR‐B‐92, and CAR‐DK‐92 cell lines. The cytotoxic effects of CAR‐NK‐92MI, CAR‐B‐92, and CAR‐DK‐92 on (F) CD19‐K562 and (G) NALM6 for 20 h under different *E:T* ratios, with *n* = 3 for each measurement. Statistical analysis was performed using Two‐way ANOVA. Data statistics are presented as mean ± standard deviation (SD). Significance levels are indicated as follows: *p < 0.05, **p < 0.01, ***p < 0.001, ****p < 0.0001.

CAR signalling has been shown to induce STAT3 activation [29]. To further examine the impact of B2M KO on the functionality of CD19‐CAR‐NK‐92MI, we constructed both CD19‐CAR‐NK‐92MI (CAR‐NK‐92MI) and CD19‐CAR‐B‐92 (CAR‐B‐92) (Figure 6A). In comparison to the B‐92 cells, the cytotoxic efficiency of the CAR‐B‐92 cells was significantly enhanced. Importantly, there was no significant difference in cytotoxic efficiency between the CAR‐B‐92 and CAR‐NK‐92MI cells (Figure 6B,C). Furthermore, we developed CD19‐CAR‐DK‐NK‐92MI (CAR‐DK‐92) (Figure 6D), which also demonstrated no significant difference in cytotoxic efficiency when compared to the CAR‐NK‐92MI and CAR‐B‐92 cells (Figure 6F,G). To verify whether CAR activation plays a role through the STAT3 pathway, we stimulated CAR‐B‐92, CAR‐DK‐92, and CAR‐NK‐92MI with 10 μg/mL Recombinant Human CD19 Protein and subsequently performed Western blot detection. The results indicated that CAR stimulation increased STAT3 phosphorylation levels of CAR‐B‐92 (Figure 6E).

### 3.6 PTPN6/B2M Double Knockout or CAR Modification Overcome the Issue of Reduced B‐92 Cytotoxicity In Vivo

**FIGURE 7:**
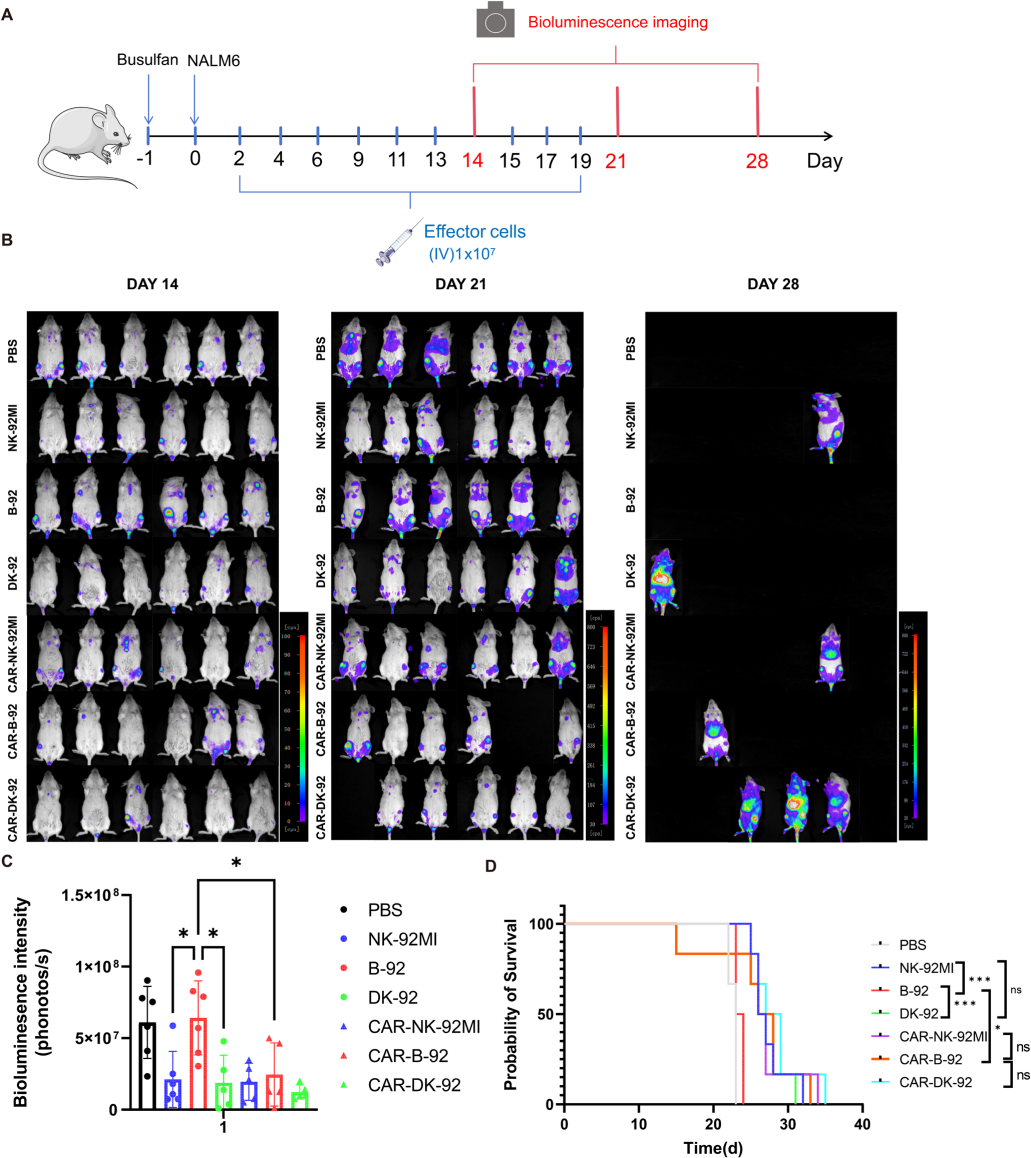
The cytotoxic efficiency of NK‐92MI, B‐92, DK‐92, CAR‐NK‐92MI, CAR‐B‐92, and CAR‐DK‐92 against NALM6 in vivo. (A) Schematic diagram of the treatment regimen for validating the efficiency. (B) Representative bioluminescence images of each group collected on indicated days. (C) Statistical analysis of bioluminescence intensity. (*n* = 6, one‐way ANOVA). (D) Kaplan–Meier survival curves for mice (*n* = 6, log‐rank test). Data statistics are presented as mean ± standard deviation (SD). Significance levels are indicated as follows: *p < 0.05, **p < 0.01, ***p < 0.001, ****p < 0.0001, and ns for *p* > 0.05.

To assess the in vivo cytotoxic activity of B‐92, DK‐92, and CAR‐B‐92, we established an NCG mouse tumour model using CD19‐positive NALM6‐luciferase cells. We then administered six types of effector cells (NK‐92MI, B‐92, DK‐92, CAR‐NK‐92MI, CAR‐B‐92, CAR‐DK‐92) and PBS as controls (Figure 7A). The growth of tumour cells in the mice was dynamically monitored by bioluminescence imaging. At day 21, the bioluminescence imaging analysis revealed that the tumour burden of mice infused with B‐92 was significantly higher than that of other effector cells. The intensity of the bioluminescence signal of the NK‐92MI, DK‐92, CAR‐NK‐92MI, CAR‐B‐92, and CAR‐DK‐92 groups was comparable (Figure 7B,C). Median survival times of the PBS, NK‐92MI, B‐92, DK‐92, CAR‐NK‐92MI, CAR‐B‐92, and CAR‐DK‐92 groups were 23.0, 26.5, 23.5, 26.5, 26.5, 27.0, and 28.0 days, respectively. Compared with B‐92 groups, DK‐92 and CAR‐B‐92 groups significantly prolonged the survival of mice (Figure 7D). Collectively, the above results proved that B2M knockout impaired the in vivo anti‐tumour ability of NK‐92MI cells, while PTPN6 knockout or CAR modification rescued B‐92 function.

## 4 Discussion

Various human NK cell lines have been established over the past few decades, including NK‐92, HANK‐1, KHYG‐1, NK‐YS, NKG, YT, YTS, NKL, and NK3.3 [4, 30]. NK‐92MI is an IL‐2‐independent variant of NK‐92, which has been the focus of extensive research and has been evaluated in clinical trials [31]. However, since NK‐92MI cells are allogeneic by nature, the development of universal cell therapy products based on NK‐92MI still requires strategies to address clinical application needs.

Alloreactive T lymphocytes are thought to recognise polymorphic residues on allogeneic MHC‐I molecules regardless of the bound peptide [32]. B2M plays a crucial role in stabilising MHC‐I molecules on the cell surface, and numerous studies have shown that eliminating B2M, which results in MHC‐I deficiency, can effectively mitigate immune rejection in allogeneic transplantation [33, 34, 35]. B2M knockout has been used to generate universally compatible stem cells with normal self‐renewal, multilineage differentiation, and tissue repair and regeneration capacities [22, 36]. Additionally, the simultaneous knockout of B2M, CIITA, and TRAC represents a viable strategy for producing universal CAR‐T cells, with triple‐knockout T cells showing better persistence in vivo compared to HLA‐sufficient T cells [37]. In contrast to T cells, a notable advantage of allogeneic NK cells is their safe administration without the risk of GVHD, and the B2M knockout strategy has been employed to develop safer and more efficient ‘off‐the‐shelf’ cellular NK cell therapies [38].

Unlike stem cells or T cells, whose functions are unaffected by the loss of MHC‐I, the functionality of NK cells is influenced by the presence of MHC‐I, as proposed by the ‘licensing’ hypothesis. MHC‐I downregulation can induce either NK cell tolerance or killing, depending on the context [39]. Studies have reported on the knockout of the B2M gene in NK cells derived from human peripheral blood, induced pluripotent stem cells (iPSC)‐induced NK cells, and embryonic stem cells (ESC)‐induced NK cells [38, 40, 41]. Several studies have indicated that either the overexpression of HLA‐E or the genetic ablation of adhesion ligands is essential for protecting MHC‐I‐negative NK cells from fratricide and for engineering resistance to rejection [38, 42]. Conversely, it has also been reported that B2M knockout in pluripotent stem cells does not affect their differentiation into NK cells nor the functionality of the differentiated NK cells [40]. However, according to the data published by the researchers, the expression of activating and inhibitory receptors of NK cells derived from B2M‐KO‐ESC was significantly altered following B2M knockout. Additionally, research has demonstrated that NK cells can adapt to the global downregulation of MHC‐I and exhibit tolerance to missing‐self [39]. However, the effects of B2M knockout on human NK cells from various sources may differ. This study reveals the negative effects of B2M knockout on NK‐92MI cell lines and explores the related molecular mechanisms to support the development of universal cell therapy products based on NK‐92.

NK‐92MI cells express numerous activating receptors, including NKp30, NKp46, and NKG2D, while presenting low levels of KIR2DL4 and lacking most inhibitory killer cell immunoglobulin‐like receptors (KIRs) [8, 43]. Additionally, NK‐92MI cells express other inhibitory receptors, such as immunoglobulin‐like transcript 2 (ILT‐2) and NKG2A/CD94 [44]. This unique profile equips NK‐92MI cells with potent cytotoxicity against MHC‐I‐positive cancer cells, which is different from PB‐NK. However, B2M deletion results in a reduction of activating receptors, cytokine production, and cytotoxicity, while also altering the transcriptional signature in B‐92 cells, which resembles the characteristics of unlicensed NK cells. Strikingly, STAT3 directly regulates the transcription of multiple activating receptors and anti‐apoptotic proteins in NK cells [45, 46, 47].

**FIGURE 8:**
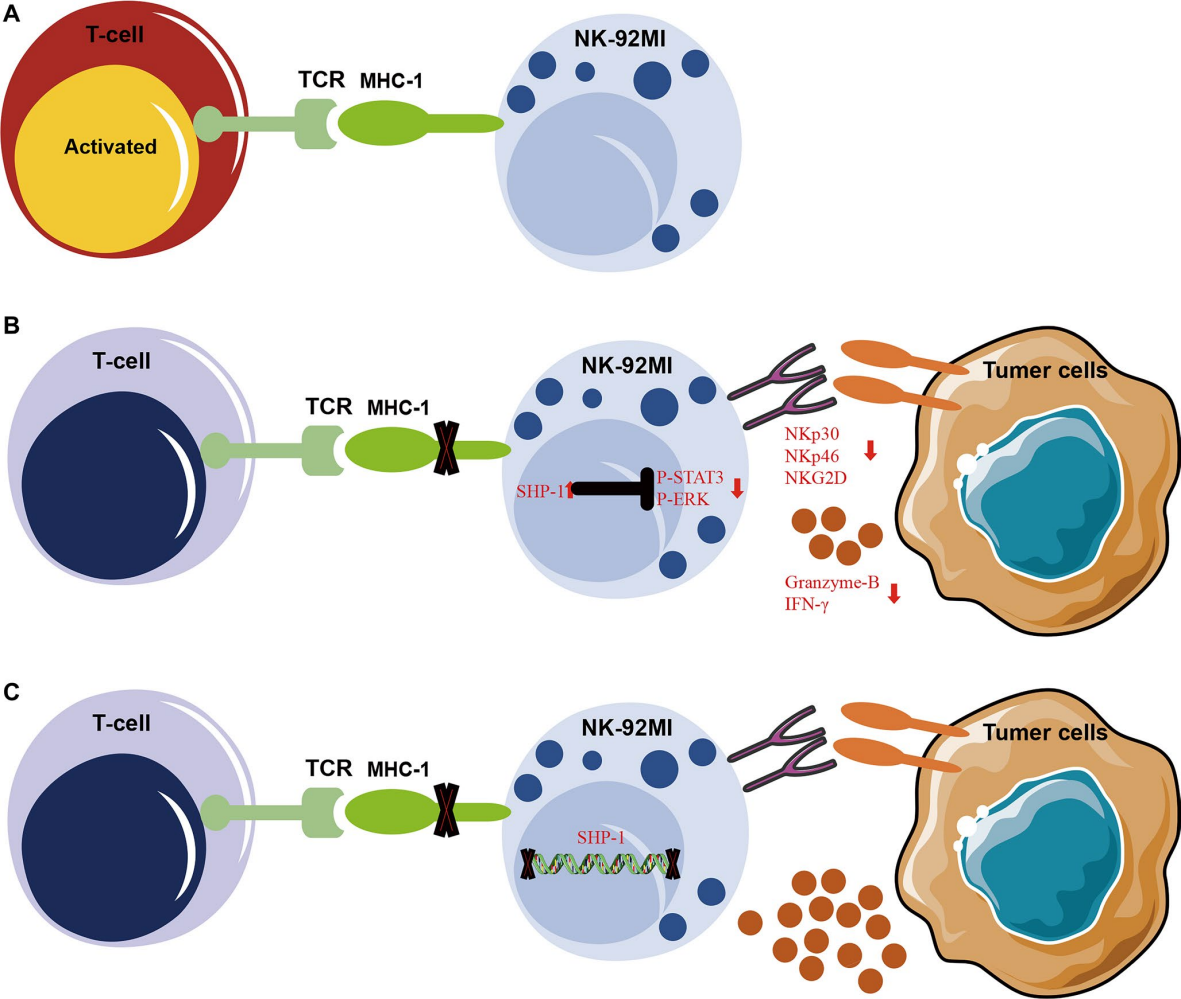
Schematic diagram illustrating the effects of B2M knockout on the biological functions of NK‐92MI and the restoration of these functions. (A) The allogeneic infusion of NK‐92MI induces immune rejection issues, activating T cells that subsequently attack the infused NK‐92MI cells. (B) The knockout of the B2M gene in NK‐92MI abrogates MHC‐I expression on the cell membrane, preventing binding with T cell receptors (TCRs) and protecting against T cell‐mediated attacks. B2M knockout leads to an increase in phosphorylation level of SHP‐1, decreased phosphorylation of STAT3 and ERK1/2, suppression of cell surface activating receptors, and reduced production levels of Granzyme B and INF‐γ. (C) Concurrent knockout of both B2M and SHP‐1 does not provoke T cell attacks while maintaining normal cytotoxic function.

Through a rescue experiment, the restoration of the NK‐92MI phenotype and function further emphasises the essential role of self‐MHC‐I in the functional education of NK‐92MI cells. Consistent with recent findings, SHP‐1 expression and phosphorylation levels are increased in MHC‐I‐deficient NK cells. SHP‐1 catalyses the dephosphorylation of tyrosine sites on ERK and STAT3, which decreases the expression of activating receptors and weakens NK cell activation [48]. Notably, PTPN6/B2M double knockout can significantly reverse the phenotype, cytokine production, and cytotoxicity of MHC‐I‐deficient NK‐92MI cells. Here, we demonstrate that B‐92 cells become hyporesponsive primarily through the SHP‐1/STAT3/ERK signalling pathway (Figure 8).

Interestingly, we observed that CAR‐NK‐92MI exhibits strong killing ability against target cells despite its MHC‐I deficiency. B2M knockout leads to an increase in SHP‐1, while CAR activation upregulates the phosphorylation of several key proteins, including STAT3 and ERK [29]. CAR modification overcomes SHP‐1‐mediated inhibition by promoting STAT3 phosphorylation, enabling B‐92 to activate normally and perform its biological functions. However, it is important to note that tumours are highly heterogeneous. The combination of PTPN6/B2M double knockout with CAR modification represents a more promising alternative for generating universal NK‐92 therapeutics, as it enhances both CAR‐dependent and CAR‐independent targeting capacities. However, further investigations are needed in the development of therapeutic products to prevent rejection by patient NK cells, as B2M knockout NK‐92MI cells are easily cleared by recipient NK cells.

## 5 Conclusion

Our research demonstrates that the knockout of B2M results in a reduction of NK‐92MI cytotoxicity. Furthermore, the phosphorylation of STAT3 and ERK is inhibited in a SHP‐1‐dependent manner. By employing either a PTPN6/B2M double knockout or CAR modification, NK‐92MI can maintain low immunogenicity while preserving its normal cytotoxic function.

## Author Contributions

**Kuo Yu:** writing‐original, draft; visualisation, validation; methodology, investigation, formal analysis, data curation, conceptualization. **Xiaolong Liu:** writing‐review and editing, validation, methodology, investigation, conceptualization. **Guangyuan Wu:** validation, software, resources, investigation, formal analysis, data curation. **Zhongyao An:** investigation, methodology. **Xin Wang:** investigation, methodology. **Yang Liu:** investigation, methodology. **Hailong Wang:** investigation, methodology. **Mingli Huang:** resources. **Linlin Zhao:** resources, supervision, software. **Ce Shi:** resources, software, supervision. **Xin Sun:** methodology. **Lu Xu:** methodology. **Sen Qi:** resources. **Xin Zhang:** resources. **Yueqiu Teng:** resources, software. **Song Guo Zheng:** writing‐review and editing, resources. **Zhiren Zhang:** resources, funding, supervision. **Zhenkun Wang:** writing‐review and editing, validation, supervision, resources, project administration, investigation, funding acquisition, conceptualization.

## Ethics Statement

The authors have nothing to report.

## Conflicts of Interest

The authors declare no conflicts of interest.

## Data Availability

The data that support the findings of this study are available from the corresponding author upon reasonable request.
